# The ECG Vertigo in Diabetes and Cardiac Autonomic Neuropathy

**DOI:** 10.1155/2011/687624

**Published:** 2011-05-29

**Authors:** Christina Voulgari, Nicholas Tentolouris, Christodoulos Stefanadis

**Affiliations:** ^1^Department of Propaedeutic and Internal Medicine, Athens University Medical School, Laiko General Hospital, 17 Saint Thomas Street, Athens 11527, Greece; ^2^Department of Cardiology, Athens University Medical School, Hippokration Hospital, 114 Vasilissis Sofias Avenue, Athens 11527, Greece

## Abstract

The importance of diabetes in the epidemiology of cardiovascular diseases cannot be overemphasized. About one third of acute myocardial infarction patients have diabetes, and its prevalence is steadily increasing. The decrease in cardiac mortality in people with diabetes is lagging behind that of the general population. Cardiovascular disease is a broad term which includes any condition causing pathological changes in blood vessels, cardiac muscle or valves, and cardiac rhythm. The ECG offers a quick, noninvasive clinical and research screen for the early detection of cardiovascular disease in diabetes. In this paper, the clinical and research value of the ECG is readdressed in diabetes and in the presence of cardiac autonomic neuropathy.

## 1. Introduction

T he importance of diabetes, both type 1 and type 2, in the epidemiology of cardiovascular diseases cannot be overemphasized. About one third of acute myocardial infarction patients have diabetes, the prevalence of which is steadily increasing [1]. The decrease in cardiovascular mortality in people with diabetes is lagging behind compared to that of the general population [2]. Cardiovascular disease is a broad term which includes any condition causing pathological changes in blood vessels, cardiac muscle or valves, and cardiac rhythm. Diabetic cardiomyopathy, although a part of the diabetic atherosclerosis process, is a distinct clinical entity and may be independent of the coexistence of ischemic heart disease, arterial hypertension, or other macrovascular complications [3]. Its pathological substrate is characterized by the presence of myocardial damage, disturbance of the management of the metabolic cardiovascular load, and cardiac autonomic neuropathy (CAN) [4]. These alterations make the diabetic heart susceptible to ischemia and less able to revive from an ischemic attack.

To philosophers, the question about which came first, the chicken or the egg evoked the questions of how life and the universe in general began [5]. Translating this causality dilemma in the language of “cardiovascular diabetology,” from one hand, diabetes is considered an equivalent to coronary heart disease, and from the other hand, coronary heart disease accounts for 65% to 80% of deaths in patients with diabetes [6]. However, the merits of screening clinically asymptomatic patients with diabetes for either the presence of coronary atherosclerosis or silent myocardial ischemia remain controversial [7]. Some observers advocate for the application of noninvasive screening testing only among patients with diabetes in whom the diagnosis of coronary heart disease is highly probable due to the presence of significant clinical coronary heart disease risk factors [8]. Thus, noninvasive screening testing will probably serve in the identification of patients with diabetes and serious coronary obstruction, in which coronary revascularization and optimum medical therapy may be considered in order to achieve the avoidance of a fatal cardiac event. Nevertheless, another physician observational group sustains the efficacy of cardiovascular testing in clinically asymptomatic people with diabetes, exactly because of either the presence of atypical symptoms or the absence of symptoms often encountered in the presence of a pathophysiological substrate equal to coronary heart disease, which, compared to the general population, differentiates significantly the medical approach in patients with diabetes [9]. 

Over the past decades, the 12-lead classic ECG has maintained its special significance for the diagnosis and triage of patients with suspected coronary heart disease and has been widespread utilized both in the diagnostic and the researcher quest as a detection and screening tool of myocardial injury. In the present paper, we aimed to summarize the ECG signs and patterns in terms of their relevance firstly to the clinician to help with the everyday diagnosis, screening, and timely decision making, but also secondly in terms of their application in the future and ongoing research. For this purpose, the medical literature on ECG manifestations of diabetes and cardiac autonomic neuropathy was systematically searched. We used the PubMed and Embase databases up to January 2011 using the following keywords: “diabetes,” “cardiomyopathy,” “cardiac autonomic neuropathy,” “neuropathy,” “autonomic dysfunction,” “electrocardiography,” “vectorcardiography,” “QT interval,” “spatial QRS-T angle,” “pattern,” “signs,” “markers,” “diagnosis,” and “treatment” alone and in combination to retrieve available literature data. All types of articles (randomized controlled trials, original studies, review articles, case reports) in humans and animals published in English, German, French, and Romanian language were included. Publications were studied in full. 

## 2. The ECG as a Diagnostic Tool in Diabetic Cardiomyopathy

### 2.1. The ECG Pattern in Early Diabetic Cardiomyopathy

In the everyday clinical practice, the ECG offers a quick, noninvasive clinical and research screen for the early detection of cardiovascular disease. Reactive hypertrophy, intermediary fibrosis, and structural and functional changes of the small coronary vessels, especially in the basal area of the left ventricle, have frequently been observed in patients with diabetes and diabetic cardiomyopathy, even when cardiac involvement is clinically not yet evident [10]. 

The preclinical phase of diabetic cardiomyopathy may be diagnosed by demonstrating exercise-induced left ventricular dysfunction, even when the resting cardiac function is still adequate [11]. The early stage of diabetic cardiomyopathy may already be associated with a range of metabolic abnormalities and even with abnormalities in diastolic function [12]. However, frequently no structural cardiac abnormalities can be identified at this stage, and the often subtle ECG alterations may be the only way to diagnose early diabetic cardiomyopathy. 

**Figure 1:**
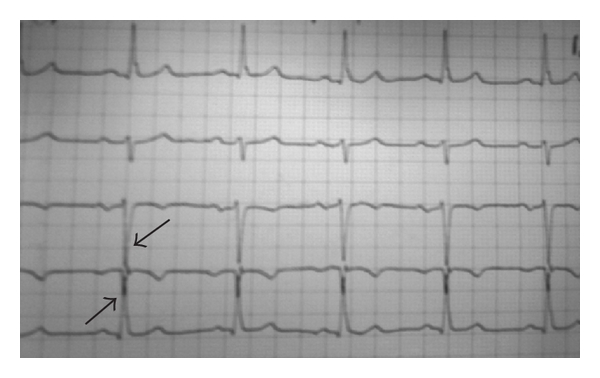
A 45-year-old man underwent routine blood tests that revealed a fasting blood glucose value of 125 mg/dL and hemoglobin A1c of 6.5%, resulting in the diagnosis of type 2 diabetes. Resting 12-lead ECG showed deep S-wave in Lead III and R-wave in Lead aVL, indicating early left ventricular hypertrophy. Cardiac autonomic functional testing diagnosed the presence of cardiac autonomic neuropathy. Stress ECG demonstrated a 2-mm depression of the ST segment. Transthoracic 2D Doppler echocardiography performed revealed presence of mild left ventricular (LV) hypertrophy (LV wall mass index = 126 g/m^2^) and an abnormal relaxation pattern (E/A < 1) with preserved LV systolic function (LV ejection fraction >60%). The patient was given strict diet restrictions; oral antidiabetic medication, b-blocker, statin and aspirin therapy was initiated, together with lifestyle measures to control cardiovascular risk factors. Throughout a 6-year follow-up, his diabetes remains well controlled, and the ECG unchanged.

Resting 12-lead ECG may show alterations of P-wave indexes (i.e. increased P-wave duration, prolongation of the PR interval, and enlarged P-wave terminal force), indicating besides atrioventricular conduction alterations, and arrhythmia, increased accumulated pericardial fat [13], which attributes to the progression of coronary atherosclerosis and is an independent risk factor for stenotic coronary artery disease [14]; increased Cornell voltage [15, 16] or Sokolow-Lyon voltage and left ventricular strain pattern [17, 18] may also be present indicating early left ventricular hypertrophy.  Figure 1 illustrates an example of the ECG pattern in early diabetic cardiomyopathy with presence of left ventricular hypertrophy in a patient newly diagnosed with type 2 diabetes. 

### 2.2. The ECG Pattern in Silent Myocardial Ischemia and Its Prognostic Value

Silent myocardial ischemia is a common, underrecognized condition that is associated with an adverse prognosis. It is a marker of significant underlying coronary heart disease and, therefore, of future cardiovascular events [19]. Silent myocardial infarction is more prevalent in type 2 diabetes and occurs in greater than one in five clinically asymptomatic patients with type 2 diabetes [20]. Resting ECG abnormalities, erectile dysfunction, peripheral vascular disease, and cardiac autonomic neuropathy are among the main clinical markers predictive of silent myocardial ischemia, and their presence should prompt further investigation in clinically asymptomatic patients with diabetes [21]. Carotid or peripheral arterial disease, proteinuria, male gender, age > 60 years, and presence of two or more of the following cardiovascular risk factors: smoking, microalbuminuria, dyslipidemia, hypertension, a family history of premature cardiac disease, and cardiac autonomic neuropathy, have been demonstrated to be the best current predictors of silent myocardial ischemia and silent coronary arterial stenosis [22]. New markers, such as adhesion molecules, Lipoprotein(a), inflammation parameters or homocysteine, osteoprotegerin and markers of endothelium function assessment might also be of further help in the future [22, 23].

A recent 5-year follow-up study in patients with type 2 diabetes, aged 50–75 years, without known or suspected coronary heart disease, compared traditional, inflammatory and prothrombotic emerging cardiac risk factors with presence of CAN as a predictor for silent myocardial ischemia. Silent myocardial ischemia was diagnosed by stress testing, and presence of CAN was the strongest predictor of ischemia in both sexes together with male gender and duration of diabetes. Among the ECG markers, ischemic adenosine-induced ST-segment depression with normal perfusion was associated with a 3-fold increased risk of silent myocardial ischemia in women [24]. 

**Figure 2:**
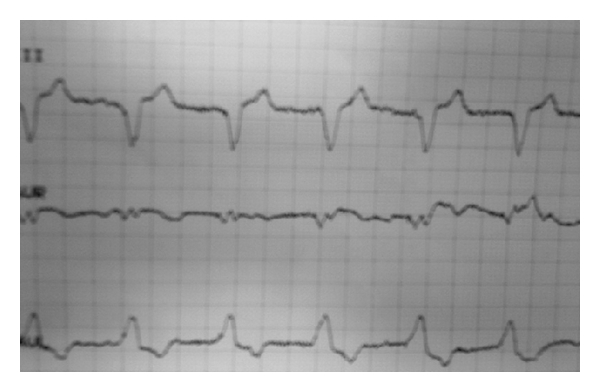
Presence of silent myocardial ischemia in the resting ECG of a middle-aged (50 years), clinically asymptomatic female patient with type 2 diabetes. Mean duration of diabetes was 4 years and the patient was under oral antidiabetic medication with an HbA1c <7%. The patient was obese (BMI > 30 kg/m^2^), with central fat distribution (waist circumference > 88 cm), without a history of hypertension, or of coronary heart disease, but with dyslipidemia and presence of microalbuminuria. She smoked 25 packs of cigarettes/year. Cardiac autonomic functional testing revealed increased cardiac sympathetic activity and parasympathetic withdrawal.

Isolated minor nonspecific ST-segment and T-wave abnormalities, indicating increased risk for coronary heart disease mortality and primary arrhythmic death can be present in the resting ECG in elderly (≥70 years) patients with diabetes [25]. Exercise microvolt T-wave alternans identify sudden cardiac death risk and have been identified in the ECGs of postmyocardial infarction patients with diabetes [26]. Computerized ECG measures of repolarization abnormality and complexity, that is, ST-segment depression ≥50 *μ*V, QT interval corrected for heart rate (QTc) >460 ms, and increased principal component analysis (PCA) of the ratio of the second to first eigenvalues of the T-wave vector (PCA ratio) can predict silent myocardial ischemia and all-cause mortality in patients with type 2 diabetes [27]. Finally, unrecognized Q-wave myocardial infarction can be present in patients with type 2 diabetes, hypertension, and microvascular complications [28].  Figure 2 illustrates the presence of silent myocardial ischemia in the ECG pattern of a clinically asymptomatic patient with type 2 diabetes and cardiac autonomic dysfunction.

Diabetes was recently demonstrated as the strongest predictor of atrial fibrillation progression and patients with diabetes and CAN frequently have asymptomatic episodes of atrial fibrillation with silent arrhythmia progression [29]. Moreover, patients with type 2 diabetes and atrial fibrillation may be at substantially higher risk of death of any cause compared with those without atrial fibrillation or without diabetes [30]. Therefore, atrial fibrillation pattern in patients with diabetes should be regarded as a prognostic marker of adverse outcome and prompt aggressive management of all risk factors is required [30]. 

In clinically asymptomatic patients with type 2 diabetes and a normal resting ECG, exercise testing is the first choice for screening for silent myocardial ischemia, whereas thallium scintigraphy with dipyridamole can be performed if exercise testing is not possible or is inconclusive [31]. The accuracy of stress ECG is 79% compared to coronary arteriography which is considered as the screening golden standard [32]. By combining stress ECG with myocardial scintigraphy, the detection of more patients with diabetes and coronary artery stenoses can be effected and a higher prediction of cardiovascular events (95.4%) achieved. However, the ECG stress test has a good negative predictive value for cardiac events (97%), it is cheaper, and should, therefore, be proposed first [33].

Ischemia ECG-stress test pattern is among the significant predictors of cardiac events (4-fold increased risk in abnormal response), and myocardial scintigraphy has an equal predictive value, followed by presence of peripheral or carotid occlusive arterial disease (10-fold increased risk) and finally coronary stenoses (27-fold increased risk in their presence) [34]. One recent prospective study examined the association between exercise ECG responses and mortality in 2.854 men (mean age 50 years) with diabetes who had previously completed a maximal treadmill exercise test and who did not have cardiovascular disease history at baseline. During a 16-year follow-up an abnormal and even equivocal exercise ECG response was associated with a statistically significant 2-fold higher risk for all-cause and cardiovascular morbidity and mortality, independently of physical fitness and other traditional cardiac risk factors, such as hyperglycemia, smoking, body mass index, hypercholesterolemia, hypertension, and a family history of cardiovascular disease or diabetes. Fit men had a higher survival rate than did unfit men [35].

The use of screening before an exercise training program for asymptomatic patients with type 2 diabetes is considered justifiable [36]. Several recent prospective studies have addressed the value of screening for coronary heart disease in clinically asymptomatic patients with diabetes [25, 37, 38]. The overall message of these studies is that despite detection of silent myocardial ischemia in a notable proportion of patients with diabetes, the dynamic pathophysiological and clinical nature of myocardial ischemia, the prohibitive cost of screening all asymptomatic patients, and the proven efficacy of primary preventive strategies would finally mandate implementation of better clinical risk stratification strategies for identifying increased at-risk individuals. However, the best strategy that would allow proper patient selection through logical stepwise approaches to screening still remains to be determined. Whether that strategy would alter patients outcome when added to rigorously implement primary preventive measures also waits to be answered. 

### 2.3. QT Interval as an Endpoint in Diabetes Trials and Its Prognostic Value

From QT interval parameters, both the QT dispersion (calculated as the mean difference between the QT maximum and the QT minimum interval in all ECG leads) and the QTc maximum have been demonstrated as independent predictors of cardiovascular mortality (for each 10-ms increment in QT dispersion, 1.5-fold increased risk for cardiovascular mortality). Excluding patients with prior cardiac disease did not change significantly the prognostic performance of QT dispersion but decreased that of QTc maximum [39]. An earlier study also found QT dispersion to be the most important independent predictor (3-fold increased risk) of total mortality and also an independent predictor of cardiac and cerebrovascular mortality [40]; however, these observations were not further confirmed in a later study. 

QTc interval >460 ms has been associated with a 2-fold increased cardiovascular and all-cause mortality risk in a 5-year prospective population study in individuals with type 2 diabetes. ST-segment depression ≥2 mm and a PCA ratio of the T-wave vector >32% in women and >25% in men were also independent predictors of mortality (4-fold and 3-fold increased risk for each ECG alteration, resp.) [41]. 

A significant correlation has been demonstrated between the QT interval duration and the amount of coronary calcium measured by coronary artery calcified plaque; this association was mainly driven by the prolongation of the QRS interval and not of the JT interval duration. The association between abnormal myocardial repolarization and coronary artery disease was found to be more significant in men than in women with diabetes [42]. 

### 2.4. The ECG Pattern in Type 1 Diabetes and CAN: Similarities and Differences

In type 1 diabetes, decreased parasympathetic to increased sympathetic tone ratio, clinically manifested by supraventricular tachycardia, and electrocardiographically evidenced by the shortening of the ventricular activation time, as well as differences in the depolarization and the repolarization ECG-pattern, have been revealed with increased QT and QT dispersion intervals, as well as with the shortening of the QRS interval [43]. Recently, it was demonstrated that during spontaneous hypoglycemia in patients with type 1 diabetes, QTc interval can be moderately increased [44], whereas glucose-QTc association is reported to differ among individuals with type 1 diabetes. Moreover, hypoglycemia unawareness was demonstrated to be the only independent predictor of the glucose-QTc association's individual strength in patients with type 1 diabetes [45].

In the Europe and Diabetes (EURODIAB) study on patients with type 1 diabetes with a normal QTc interval at baseline, the cumulative incidence of prolonged QTc interval after the 7-year follow-up was 19% and was 2-fold higher in women than in men. Seven-year incidence of QTc prolongation was associated at baseline with older age, physical inactivity, arterial hypertension, dyslipidemia (assessed by lower high-density lipoprotein cholesterol levels), presence of peripheral neuropathy, and coronary heart disease. Independent predictors of increased QTc-prolongation risk were female gender, higher values of glycated hemoglobin A1c (HbA1c), and systolic blood pressure levels, whereas physical activity and normal body mass index were the main protective factors [46]. 

In the WHO Multinational Study of Vascular Disease in Diabetes in Switzerland, during a follow-up duration of 23 years, 523 patients with diabetes (221 with type 1 diabetes, and 302 patients with type 2 diabetes) were studied and the association of QTc interval with total, cardiovascular, and ischemic heart disease mortality was evaluated. Different results were noted among the studied patients. In subjects with type 1 diabetes, QTc was associated with an increased risk of all-cause mortality (hazard ratio: 1.2-fold increased risk per 10 ms increase in QTc interval) and mortality due to cardiovascular disease. Findings for subjects with type 2 diabetes were different: only increased heart rate and not QTc interval was associated with total mortality, and mortality due to cardiovascular, as well as ischemic heart disease (hazard ratio: 1.3-fold increased risk per 10 beats increase per min). Therefore, QTc interval was significantly correlated with long-term mortality in subjects with type 1 diabetes, whereas it was not related with increased mortality risk in subjects with type 2 diabetes [47]. QTc interval was considered as a marker of cardiac autonomic dysfunction, and this difference between the two types of diabetes was attributed to the greater importance of CAN noted in patients with type 1 rather than in patients with type 2 diabetes [48]. However, it may also be related to the recently described “electromechanical window” which portrays the temporal difference between the electrical and mechanical events of the heart and has currently been demonstrated as comprising a potential predictive value for the occurrence of arrhythmias in cardiac autonomic dysfunction [49].

The EURODIAB Insulin-Dependent Diabetes Mellitus Complications Study (EURODIAB IDDM) investigated 3.250 patients with type 1 diabetes and average diabetes duration of more than 30 years; the prevalence of left ventricular hypertrophy was found to be 3 times greater than that reported in the general population of similar age. Moreover, the prevalence of left ventricular hypertrophy was 2-fold higher in women than in men, independent of well-known cardiovascular risk factors, that is, arterial hypertension, obesity, and physical inactivity. The 2-fold higher risk of left ventricular hypertrophy in women was significantly associated with higher serum triglycerides, central obesity (waist circumference > 88 cm), hypertension, and presence of coronary heart disease. Moreover, prolongation of the QTc interval was significantly associated with left ventricular hypertrophy prevalence in both sexes; however, this was not further validated for QT dispersion [50]. 

In the Losartan Intervention For Endpoint reduction in hypertension (LIFE) study of 9.000 patients with hypertension, but without diabetes, during the 5-year follow-up, regression or persistent absence of left ventricular hypertrophy on the ECG pattern during antihypertensive treatment was associated with a lower rate of new-onset diabetes (38%). Even after adjustment for the new diabetes onset with prior antihypertensive treatment, baseline glycemia, the Framingham risk score, baseline and in-treatment blood pressure levels, high-density lipoprotein cholesterol, uric acid, and body mass index, and the decreased incidence of diabetes onset associated with losartan-based therapy (26% lower risk of new diabetes onset), which occurred in parallel with the in-treatment left ventricular hypertrophy amelioration or absolute resolution, remained statistically significant [51]. 

### 2.5. The Genetic Pathophysiological Substrate of the ECG in Diabetes and CAN

Genetic variants in myocardial sodium and potassium channel genes in previously identified candidate genes may be associated with the QT interval duration, the presence of cardiovascular autonomic dysfunction, and the increased risk of sudden cardiac death in individuals with diabetes [52]. 

### 2.6. Vectorcardiography: New Markers in the Aid of the ECG Diagnosis of Diabetic Cardiomyopathy

The spatial QRS-T angle is the ECG integral of the heart's ventricular gradient [53], which quantifies the deviation between the directions of the ventricular depolarization and repolarization [54]. The spatial QRS-T angle represents a global measure of the variations of ventricular action potential durations and morphology and, therefore, serves as a reliable and easy to measure with modern electrocardiographs ECG marker of arrhythmia vulnerability [55]. Due to its electrophysiological substrate, the spatial QRS-T angle has been proven to be a more sensitive and of higher cardiovascular predictive value marker than the QT interval and its parameters [56, 57]. 

Spatial QRS-T angle values were demonstrated to be higher (by almost 2-fold) in patients with diabetes compared to patients without diabetes. Higher spatial QRS-T angle values were also independently associated with glycemic control and impaired left ventricular myocardial performance in patients with type 2 diabetes [58]. Moreover, in patients with type 1 diabetes during hypoglycemia, where the QTc interval or the QT dispersion remained unimpaired, the morphology of the spatial QRS-T angle changed significantly, indicating increased arrhythmia vulnerability. The increase in the spatial QRS-T angle values was independent from any changes in catecholamine levels or in the heart rate of the patients with type 1 diabetes [59]. The prognostic importance of the higher spatial QRS-T angle values in subjects with diabetes, however, remains to be evaluated in future prospective studies.  Table 1 summarizes major and recent studies addressing the clinical significance of the ECG markers in the diagnosis of Diabetic Cardiomyopathy and Silent Myocardial Ischemia in both type 1 and type 2 diabetes. 

## 3. Cardiac Autonomic Neuropathy in Diabetes: The “Queen's Mirror” of Cardiovascular Morbidity and Mortality

Physiological cardiovascular activities are under the control of the cardiac autonomic nervous system. Damage to the autonomic nerves that innervate the heart and blood vessels results in dysfunction in heart rate control and vascular dynamics, in other words in CAN [60]. Autonomic imbalance between the sympathetic and parasympathetic nervous systems' regulation of cardiovascular function contributes to metabolic abnormalities [61] and significant morbidity and mortality for individuals with diabetes [62]. CAN embraces exercise intolerance, intraoperative cardiovascular liability, orthostatic tachycardia and bradycardia syndromes, and silent myocardial ischemia [63]. All these clinical manifestations can result in life-threatening outcomes, which unquestionably associate the presence of CAN with the increased risk of cardiovascular morbidity and mortality in diabetes [64]. This is in agreement with recent published results from the Action to Control Cardiovascular Risk in Diabetes (ACCORD) trial, which demonstrated that the presence of CAN at baseline is an independent contributor to the higher cardiovascular mortality risk in both the intensive and standard glycemic arm treatment. No differential effect was found in subgroups defined by variables anticipated to have an interaction, that is, age, duration of diabetes, and previous history of cardiovascular disease [64, 65]. Furthermore, individuals with baseline CAN were 2 times more likely to die compared with individuals without CAN [65]. In addition, CAN in the presence of peripheral neuropathy was the strongest predictor of cardiovascular mortality [66]. 

CAN often coexists with peripheral neuropathy [60, 67, 68], and recent studies demonstrated that combining indexes of autonomic and peripheral neurological dysfunction, is associated with an earlier detection and probably prevention of the cardiovascular risk and mortality in diabetes [69, 70]. Finally, recently it was demonstrated that presence of CAN predicts development of large-fiber dysfunction, and may account for the high mortality rate in patients with peripheral neuropathy, emphasizing the importance of early detection of both CAN and peripheral neuropathy to address the cardiovascular risk in diabetes [71]. In the EURODIAB Prospective Complications Study, peripheral and autonomic neuropathy was among the strongest risk markers for future total and cardiovascular mortality exceeding the effect of the traditional risk factors, that is, age, obesity, hypertension, and dyslidemia [72]. 

## 4. Diagnostics Tests for the Assessment of Cardiac Autonomic Neuropathy

The Golden Standard in clinical autonomic testing remains the cardiovascular reflex tests. These tests have good sensitivity, specificity, and reproducibility and are noninvasive, safe, well standardized, and easily performed [87]. Exception is the Valsalva maneuver, which must not be performed in patients with proliferative retinopathy [88]. The most widely used tests assessing cardiac parasympathetic function are based on the time-domain heart rate response to deep breathing, the Valsalva maneuver, and postural change. Of these tests, heart rate response to deep breathing has the greatest specificity [89, 90]. Cardiovascular sympathetic function is assessed by measuring the blood pressure response to orthostatic change and the Valsalva maneuver [91, 92]. The performance of these tests should be standardized, and the influence of confounding variables such as medications, hydration, and antecedent activity should be minimized. Age normative values should be used. As already mentioned, the combination of cardiovascular autonomic tests with sudomotor function tests may allow a more accurate diagnosis of CAN [69, 70].

Among the CAN testing for clinical trials and research, the time-domain heart rate tests and the blood pressure response to postural change have the necessary reproducibility and have been previously used as trial endpoints, that is, in the Diabetes Control and Complications Trial/Epidemiology of Diabetes Interventions and Complications Study (DCCT/EDIC) [93] and in other clinical trials [94, 95]. Frequency-domain indexes obtained by applying spectral analysis to heart rate variability of short (5–7 min) and longer (24-h) electrocardiogram recordings [96] provide a measure of sympathetic and parasympathetic modulation of heart rate. Heart rate spectral power in the high-frequency region is a measure of parasympathetic modulation, while spectral power in the low-frequency region provides a measure of both sympathetic and parasympathetic modulation. The low-frequency blood pressure variability may provide a measure of sympathetic modulation. To correctly assess the significance of the different regions, respiration should be measured or controlled breathing performed. 

The Neuropad test is a modern indicator test, which its performance is based on the measurement of sweat production after exposure to dermal foot perspiration, is a valuable tool in the diagnosis of both peripheral [69] and autonomic neuropathy in patients with diabetes [70], and appears to be a very simple and useful indicator for screening patients with diabetic neuropathy [97]. The high degree of reliability and easiness of the Neuropad test suggest that it is proper even for self-testing for the identification of autonomic neuropathy [98]. 

QT interval prolongation is an independent predictor of mortality in patients with diabetes and is associated with CAN [62]. The pathogenesis of QT prolongation is multifactorial, and its correlates include female gender, nephropathy, coronary heart disease, glycemic control, systolic blood pressure, physical activity, and body mass index. In the recent ACCORD trial, assessment of CAN included heart rate (reflecting overall autonomic function and cardiorespiratory fitness), measures of heart rate variability, and the QT interval (reflecting mainly sympathetic function) computed from 10-s resting electrocardiograms [64]. 

## 5. The ECG in Cardiac Autonomic Neuropathy

### 5.1. The ECG as an Early Marker of CAN

Early markers of CAN in an ECG include elevated R-wave amplitude, prolongation of QTc interval, and decreased heart rate variability [99]. Aerobic exercise training in Zucker Diabetic Fatty rats, a model of type 2 diabetes, although it did not attenuate neither QT or QTc interval prolongation, or restored decreases in heart rate variability, however, it restored R-wave amplitude alterations and, therefore, had a beneficial effect in regards to ECG correlates of the left ventricular mass hypertrophy [73]. 

Even in healthy individuals, hyperinsulinemia-induced hypoglycemia can prolong the QTc interval and decrease T-wave area and amplitude. Counterregulatory norepinephrine response correlated with QTc interval prolongation, and epinephrine response correlated with flattening of the T-wave. Besides ECG alterations in depolarization and repolarization, hyperinsulinemic hypoglycemia with consequent sympathetic humoral activation can be associated with alterations in atrioventricular conduction and moderate ST-segment depression [76]. 

### 5.2. Associations between the ECG Markers, Metabolic and Other Factors in the Presence of CAN

Among the factors significantly associated with cardiac autonomic dysfunction are central obesity, hypertension, smoking, hyperglycemia, dyslipidemia, and presence of microvascular complications, that is, retinopathy, nephropathy, and pubertal diabetes onset in patients with type 1 diabetes. In type 2 diabetes patients, CAN has been independently associated with central obesity, hypertension, hyperglycemia, known diabetes duration, dyslipidemia, smoking, and presence of microvascular complications, as well as with peripheral vascular disease [100]. 

CAN, together with diabetes duration, has been demonstrated as the main predictor of reduced aortic distensibility in type 2 diabetes, and, therefore, it is associated with a significant reduction in the elastic properties of the aorta [101]. Reduced aortic distensibility predicts cardiovascular mortality in patients with type 2 diabetes and impaired glucose tolerance [102].

Accordingly, QTc interval prolongation has been associated with age, gender, systolic and diastolic blood pressure, body mass index, central obesity, smoking, the type and the duration of diabetes, the different types of antidiabetic medication, glycemic control, and the severity of autonomic neuropathy. Major degrees of autonomic neuropathy were found to be independent predictors of QTc interval in both type 1 and type 2 diabetes [82, 103]. These findings were recently further confirmed in a population of patients with both type 1 and type 2 diabetes and presence of CAN, where QTc interval prolongation was significantly associated with older age, longer (>10 years) diabetes duration, and the presence of peripheral neuropathy. Higher scores of cardiac autonomic dysfunction also correlated with longer QTc intervals in both type 1 and type 2 diabetes patients [78]. 

### 5.3. The Pathophysiological Substrate That Possibly Elucidates the Association of Cardiac Autonomic Neuropathy with Coronary Heart Disease Events

The increased risk of coronary heart disease events in diabetes presents an altered circadian distribution with an absent morning peak and a higher infarction rate during the evening hours. To elucidate the mechanism of this phenomenon, and the pathophysiological substrate behind increased cardiovascular risk in the presence of CAN in patients with diabetes, several studies addressed the circadian pattern of heart rate variability in diabetes complicated with CAN.

The circadian pattern of indexes of parasympathetic tone has been evaluated using 24 h heart rate variability analysis in conjunction with the circadian changes of fibrinolytic and hemostatic factors (plasminogen activator inhibitor 1, factor VII, fibrinogen, and factor von Willebrand) in patients with type 1 diabetes and presence of CAN. CAN was associated with a loss of both the nocturnal predominance of parasympathetic activity, due to a small but significant increase of the sympathetic tone during nighttime, and a prothrombotic state that persisted throughout the day [104]. These abnormalities possibly attenuate the relative protection from coronary events during the afternoon and nighttime in patients with diabetes and CAN. 

In patients with diabetes, but without CAN, a day-night modulation of the QTc interval has been evidenced and is dependent on both variations in the cardiac autonomic tone and the concentrations of circulating catecholamines [105]. This long-term modulation is significantly different in subjects with diabetes in the presence of CAN, where a circadian sympathovagal balance reverses the day-night ECG pattern and increases nocturnal QT rate dependence [106]. 

Accordingly, the circadian rhythmicity of the QT interval in patients with diabetes and varying degrees of CAN was found to exhibit a significant day-night periodicity. QTc interval was longer between midnight and early in the morning, and it was shorter in the hours after waking. These alterations were also significantly associated with the severity of CAN [107]. 

Therefore, even in patients with diabetes and mild parasympathetic denervation, QT interval heart rate dependence is impaired. These changes in cardiac electrophysiological activity may be related to the reported diurnal pattern of ventricular arrhythmias [107, 108]. The potential prognostic impact of this reversed day-night pattern with steep nocturnal QT/heart rate relation was assessed in later studies. 

### 5.4. The ECG Pattern in Cardiac Autonomic Neuropathy and Diabetes, and Its Predictive Value

The association between QT interval prolongation and the risk of dying unexpectedly in patients with diabetes and CAN has been repeatedly evaluated. Prolonged QTc interval has been significantly associated with the presence of CAN and the 3-year prevalence of sudden cardiac death in male patients, under 60 years of age, with diabetes and varying degrees of cardiac autonomic dysfunction. This relationship was independent of the effect of age and diabetes duration. QTc not only was significantly longer in the patients with diabetes who died unexpectedly, but furthermore the changes in the QT length during follow-up where parallel with the alterations in the cardiac autonomic function [83]. 

QTc interval and QT dispersion were also determined as independent predictors of cardiovascular mortality throughout a 12-year follow-up period of 192 patients with type 2 diabetes. Their predictive value was superior to that of the heart rate variation in response to deep breathing and standing and the ankle-brachial pressure index [79]. 

In the MONICA/KORA Augsburg Cohort Study, during a 9-year follow-up, reduced heart rate variability and prolonged QTc interval (>440 ms) were independent predictors of a 2-fold and 3-fold increased risk of mortality, respectively, in the general population (aged 55 up to 74 years) with or without diabetes. However, increased QT dispersion (>60 ms) did not predict mortality. From the heart rate variability parameters, low heart rate variability during spontaneous breathing tended to be associated with excess mortality in the people with diabetes, but not in those without diabetes [77]. 

In a recent case control study of 682 people with coronary heart disease and diabetes but no history of sudden cardiac death, prolongation of QTc interval was a significant predictor of sudden cardiac death even among individuals with a normal or borderline QTc interval. However, idiopathic abnormal QTc interval prolongation was associated with a higher (5-fold) increased risk of sudden cardiac death [74]. 

Nevertheless, the association of high resting heart rate, with sudden cardiac death in diabetes cannot be fully explained by the reported association between QT interval and heart rate with coronary heart disease or left ventricular dysfunction, the major pathological substrates for sudden cardiac death. Ventricular arrhythmia is the most common antecedent event before sudden cardiac death [109] and earlier [110] as well as recent studies [111] examined possible associations between resting QT interval, resting heart rate and ventricular arrhythmogenesis.

One study investigated the presence of ventricular late potentials derived from signal-averaged ECG in patients with type 1 diabetes with and without CAN, without clinical evidence of cardiovascular disease. The QTc interval was significantly prolonged in patients with autonomic dysfunction as compared with those without CAN. Moreover, in the group without CAN, there was no significant prolongation of the QTc interval. Ventricular late potentials were present in patients with diabetes and were associated with isolated presence of peripheral neuropathy but not with the presence of CAN [80]. 

However, another previous study also aimed to assess the influence of cardiovascular complications on the occurrence of ventricular late potentials in children with type 1 diabetes and an average course of diabetes duration of 6.5 years. Ventricular late potentials were discovered in 17% of the patients with type 1 diabetes and were associated with the incidence of left ventricular hypertrophy, longer duration of diabetes, and the presence of CAN. Left ventricular hypertrophy, diabetes duration, and cardiac autonomic dysfunction were also the strongest independent parameters of ventricular late potentials occurrence. The presence of cardiovascular complication had no influence on ventricular late potential occurrence in children with diabetes [112].

Finally, one recent study included 867 patients (age ≤60 years, 57% females and 17% with diabetes) who underwent 24 h ambulatory ECG recording (Holter) and also examined possible associations between resting heart rate with factors involved in ventricular arrhythmogenesis, that is, ventricular late potentials detected by signal-averaged ECG, heart rate variability, and premature ventricular complexes. High resting heart rate was significantly associated with positive ventricular late potentials, depressed heart rate variability indices, and increased prevalence of premature ventricular complexes during the 24 h recording, independently from demographic and clinical variables including left ventricular ejection fraction, and history of coronary heart disease, or presence of ST-depression in Holter analysis. Therefore, high resting heart rate and its influence on the cardiac electrophysiological cycle are independently associated with ventricular arrhythmogenesis [109]. The different results observed in this study compared with those of previous studies may be associated with the inability of the QT interval to assess primary factors that contribute to the ventricular repolarization (i.e., heterogeneity of action potential morphology throughout the ventricles) in the presence of secondary factors also contributing to the cardiac electrophysiological cycle (i.e., heterogeneity in ventricular depolarization instants) [113]. 

### 5.5. Factors Associated with Amelioration of the ECG Alterations in Cardiac Autonomic Neuropathy and Diabetes

In the Diabetes Control and Complications Trial/Epidemiology of Diabetes Interventions and Complications study (DCCT/EDIC), a prospective observational follow-up of the original DCCT type 1 diabetes cohort, cardiovascular autonomic measures (heart rate variation during deep breathing, Valsalva ratio, postural blood pressure changes, and autonomic symptoms) were repeated in 1.226 patients after 14 years of follow-up. Although, the prevalence of CAN was increased in both therapy groups, it was significantly lower in the intensive therapy versus the conventional therapy group (29% versus 35%). Heart rate variability was significantly greater (R-R interval variation >15) in former DCCT patients under intensive antidiabetic treatment versus former DCCT patients in the conventional therapy arm. 

Moreover, symptoms consistent with cardiac autonomic neuropathy dysfunction in the 13th or the 14th EDIC year, although seldom reported, were more prevalent in patients in the conventional prior DCCT therapy compared to the intensive DCCT therapy arm. In respect, among the most commonly reported symptoms, decreased adrenergic awareness of hypoglycemia was reported in 25% of the conventional versus 20% of the intensive therapy group; male impotence was determined in 23% of the prior DCCT intensive therapy versus 30% of the intensive therapy sector. Finally, excessive postprandial epigastric fullness had no significant difference between the two groups. No significant treatment group differences were found for the rest of the symptoms.

Metabolic memory was assessed for the incidence of abnormal R-R variation, abnormal Valsalva ratio and abnormal cardiac autonomic function during EDIC, in those subjects who were free of the condition at the end of the DCCT study. Prior DCCT intensive therapy reduced the risks of incident cardiovascular autonomic dysfunction by 31% and of incident abnormal heart rate variability (R-R variation <15) by 30% in patients with type 1 diabetes in the 13th or the 14th EDIC year. 

In conclusion, the benefits of intensive therapy extend to measures and symptoms of CAN up to 14 years after the DCCT closeout [75]. 

### 5.6. The ECG Pattern in Autonomic Dysfunction and Its Role in Diabetes Development in Healthy People

As previously referenced, cardiac autonomic dysfunction has been correlated with fasting insulin and glucose, independent of clinically diagnosed diabetes. In a general population prospective study, people with high resting heart rate and low heart rate variability had increased risk for future development of type 2 diabetes. In respect, participants in the uppermost (>73 beats per minute) versus the lowest (≤60 beats per minute) quartile of heart rate had a 60% increased risk of developing diabetes. Results were similar when the sample was restricted to participants with normal fasting glucose at baseline or when adjusted for baseline glucose levels. Further adjustment for age, race, gender, education, alcohol drinking, current smoking, prevalence of coronary heart disease, physical activity, and body mass index demonstrated a sustained significant relationship between cardiac autonomic dysfunction and development of diabetes [81]. 

### 5.7. The Alternative Choice of Vectorcardiography in Cardiac Autonomic Neuropathy and Diabetes

Although the classical ECG parameters has been repeatedly used for the assessment of repolarization abnormalities in diabetes and presence of CAN, they have also been frequently criticized as poor indicators of ventricular arrhythmogenicity [114, 115]. Moreover, not all studies reached in similar conclusions regarding the association between prolongation of the QT interval and presence of cardiac autonomic dysfunction [116–118]. Furthermore, calculation of the classic ECG parameters is often time consuming, subjected to methodological errors, and is also affected by heart rate [115].

As previously mentioned, the spatial QRS-T angle is a modern ECG marker aiming to assess the overall heterogeneity of ventricular action potential morphology [119]. It is obtained by vectorcardiography and can be computed on the basis of a regular routine 12-lead ECG [55]; determination of the spatial QRS-T angle has many advantages over the QT interval measurement; it can be obtained easily and faster by the newer ECGs equipment, it is not subjected to calculation errors or affected by heart-rate, it is of low cost, and it has been evaluated prospectively. 

**Figure 3:**
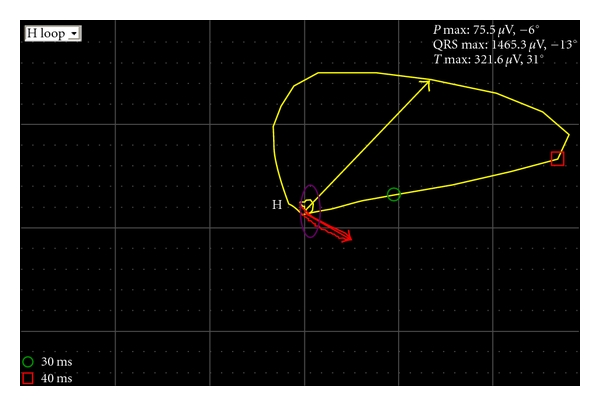
The resting vectorcardiogram of a 56-years old male subject with type 2 diabetes and cardiac autonomic neuropathy recorded with the help of a computer-based 12-lead ECG system and automatically analyzed by the Modular-ECG-Analysis program incorporated in the electrocardiograph. The amplitude of the mean spatial T-vector (red line vector) and the amplitude of the mean spatial QRS-vector (yellow line vector) form an angle of 52° (violet line between the two spatial vectors), which defines the patient's spatial QRS-T angle.

In the Rotterdam Study [56, 84], the spatial QRS-T angle was an important arrhythmogenic marker for subclinical myocardial damage. Abnormal spatial QRS-T angle values (≥105°) were found in 20% of the subjects with type 2 diabetes and were associated with increased risk of cardiac mortality and sudden cardiac death. The risks associated with a borderline spatial QRS-T angle (<75°) were also significantly increased for all outcomes. In the WISE Study [86], type 2 diabetes was associated with wider spatial QRS-T angles values (>49°), which predicted adverse cardiovascular events independently of the severity of coronary heart disease in subjects with suspected myocardial ischemia. Smaller values of spatial QRS-T angle (<49°) were also predictive of cardiovascular events in healthy individuals. Furthermore, in the Cardiovascular Health Study [85], a widening of the spatial QRS-T angle (>45°) was associated with a higher proportion (19%) of silent myocardial infarction in type 2 diabetes subjects free from coronary heart disease.  Figure 3 illustrates the vectorcardiogram of a patient with type 2 diabetes and cardiac autonomic neuropathy in the absence of QTc interval or QT dispersion prolongation as measured in the patient's resting 12-lead ECG pattern. 

One recent study demonstrated that the spatial QRS-T angle is significantly wider in subjects with type 2 diabetes and CAN [118]. Moreover, presence and severity of CAN were the strongest predictors of the spatial QRS-T angle values. Heart rate variability parameters were significantly and independently associated with the spatial QRS-T angle. The findings of this study support that preservation of the parasympathetic function in type 2 diabetes and CAN is protective, while sympathetic predominance or sympathovagal imbalance are harmful for the heart's normal electrophysiological activity and result in alterations in the spatial QRS-T angle. Additionally, from the clinical point of view, a wider spatial QRS-T angle in uncomplicated subjects with type 2 diabetes may point out to the presence of CAN, which is often underdiagnosed. 

Besides the autonomic influences on the heart's electrophysiological activity, a wider spatial QRS-T angle reflects damaged areas of the myocardium that distort the spread of electrical forces through the myocardial wall [120]. Wider spatial QRS-T angle values were also significantly and independently associated with increased left ventricular mass and worse left ventricular myocardial performance in the patients with type 2 diabetes. Noteworthy, heart rate variability parameters explained almost 50% of the spatial QRS-T angle variability, suggesting presence of a pathophysiological ground linking the structural, functional, and electrical myocardial disturbances in type 2 diabetes. These disturbances were even more exacerbated by the presence of cardiac autonomic dysfunction [118].  Table 2 summarizes major and recent studies evaluating the clinical significance of the ECG pattern in diabetes and the presence of Cardiac Autonomic Neuropathy. 

## 6. Conclusion Remarks and Thoughts

Currently ongoing research has revealed that in diabetic cardiomyopathy direct metabolic alterations of the cardiac myocyte and impaired support of extra cardiac factors regulating cardiac activity, such as sympathetic regulation of heart rate and contractility, are involved in the different alterations of the cardiac ionic currents in the heart and the provocation of arrhythmia, mainly by the reduction in potassium repolarizing currents [121, 122]. Cardiac autonomic neuropathy leads to diminished noradrenaline release in cardiac ventricle in response to different external or internal stimulus, that is, standing, exercise or stress [123]. Reduction of the cardiac myocyte response to acute noradrenaline exposure and, finally, impairment of different trophic factors responsible for the regulation of ionic channel expression due to diabetic cardiomyopathy [124] are all involved in the vulnerability and increased incidence of cardiac arrhythmia of the diabetic heart.

In this era, the ECG continues to be an invaluable tool in the initial evaluation of patients with diabetes. The plethora of data currently available on ECG changes correlating with diabetic cardiomyopathy and cardiac autonomic neuropathy allows clinicians to make faster and better decisions than ever before. Even early in the course of diabetes, ECG alterations of the rhythm, beginning from simple sinus tachycardia and moving on to different ECG parameters like the prolongation of the classical QTc interval, or the QT dispersion, and the modern wider spatial QRS-T angle deviation, ST-segment and T-wave changes, alterations in heart rate variability, and presence of left ventricular hypertrophy, may be observed. 

**Figure 4:**
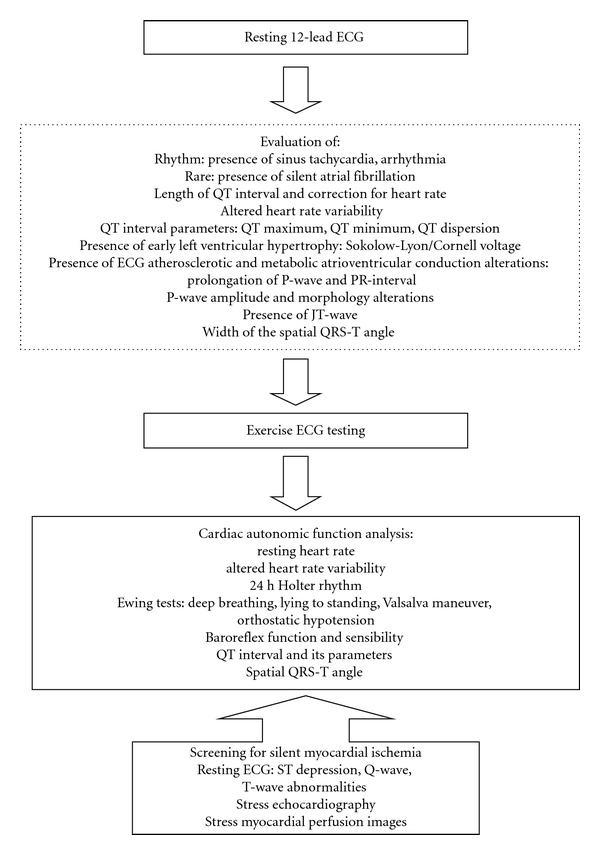
Proposed Cardiovascular Examination for Patients with Diabetes and Cardiac Autonomic Neuropathy.

ECG alterations help evaluate cardiac autonomic neuropathy and detect signs of silent myocardial ischemia even in clinically asymptomatic patients with diabetes. Prolonged myocardial fibrosis leads to diabetic cardiomyopathy, with peculiar ECG presentation. Electrocardiographic changes are often present in children and adolescents with type 1 diabetes. The resting ECG, frequently complemented by exercise ECG testing, is a major assistant in cardiac screening of individuals with diabetes and cardiac autonomic neuropathy and helps detect silent myocardial ischemia, assess prognosis, and predict cardiovascular, cerebrovascular, and all-cause mortality. As a closing remark extracted from the referenced clinical and research studies, Figure 4 summarizes a proposed cardiovascular examination for patients with diabetes and cardiac autonomic neuropathy. 

## Figures and Tables

**Table 1:** Recent and major studies of the incidence of electrocardiographic abnormalities in diabetes and their endpoint clinical significance.

Reference	Population	ECG marker	Clinical significance	Clinical points
Left ventricular hypertrophy and atherosclerosis in diabetic cardiomyopathy				

[14]	996 T2D patients	↑ 1SD P-wave duration ≥40 msec ↑ 1SD PR-interval duration ≥12 msec ↑ 1SD P-wave terminal force	↑ Pericardial fat	No association after adjustment with adiposity indexes and CVD risk factors
[15]	110 T2D patients, age 20–80 years	↑ Cornell Voltage* ↑ QRS duration	Left ventricular hypertrophy	Ongoing trial
[16]	9.193 T2D + hypertension	↑ Cornell and Sokolow Lyon Voltage	Left ventricular Hypertrophy	Hyperuricemia as a CVD risk factor
[17]	276 T2D + hypertension	↑ Cornell and Sokolow Lyon Voltage	Left ventricular Hypertrophy	↓ in LVH prevalence with candesartan
[51]	9.000 hypertensive	↑ Cornell Voltage	Left ventricular Hypertrophy	38%↓ risk of new diabetes onset
[18]	886 T2D patients	↑ Cornell and Sokolow-Lyon Voltage left ventricular strain	Left ventricular hypertrophy	Coexistence of hypertension
[42]	1.123 T2D patients	↑ QTc, ↑ QRS, ↑ JT	Coronary artery calcification	men > women

Silent myocardial ischemia and cardiovascular disease risk				

[26]	3.224 with diabetes, 61.9% women, mean age 72 years	minor nonspecific ST-segment T-wave abnormalities	↑ risk for coronary heart disease mortality ↑ risk for primary arrhythmic death	No association with incident nonfatal myocardial infarction
[27]	493 post-MI, T2D patients	↑ T-wave alternans ≥47 microV	↑ risk of sudden cardiac death	—
[35]	2.654 men, T2D patients	↓ ST segment ≥1 mm for 0.08 sec	16-y CVD and all-cause mortality	Independent of other CVD risk factors
[41]	994 T2D patients	↓ ST segment ≥50 micro V QTc > 460 ms, PCA ratio ≥30%	↑ CVD morbidity and mortality ↑ all-cause mortality	—
[28]	1.387 T2D patients	Q-wave	Clinically unrecognized MI	Coexistence of hypertension + nephropathy
[25]	1.123 T2D patients	Adenosine induced ST-depression	Silent myocardial ischemia	4-fold ↑ 5-years risk
[39]	472 T2D patients	↑ QT dispersion, QTc maximum	Prognostic marker CVD mortality	57 months follow-up ↓ prognostic value in patients without CVD
[40]	216 T2D patients	↑ QT dispersion	↑ CVD	↑ total and cerebrovascular mortality

Type 1 diabetes and diabetic cardiomyopathy				

[43]	22 T1D patients, mean age 30 years	↓ QRS <120 mse, QTc ≥450 ms, ↑ QT dispersion >70 ms	↓ parasympathetic to sympathetic tone ratio, tachycardia, shortening of the activation time	—
[46]	1.415 T1D patients	QTc >440 msec	↑ 7-year CVD risk	↑ risk in women, hypertension, hyperglycemia, CAN, ↓ risk in BMI, physical activity
[46]	3.250 T1D patients	QTc > 440 msec, QT dispersion	3-fold ↑ risk of Left Ventricular Hypertrophy	Association with female sex, obesity, hypertension, physical inactivity
[47]	523 T1D and T2D patients	↑ QTc, ↑ HR	23-years total mortality	Increased Risk for T1D: QTc; for T2D: HR
[44]	21 T1D patients	↑ QTc	Marker of spontaneous hypoglycemia	Modest increase in QTc and misleading results in investigations of spontaneous hypoglycemia

Spatial vectorcardiography in diabetic cardiomyopathy				

[58]	74 T2D patients	↑ spatial QRS-T angle	Diabetic cardiomyopathy	Association with glycemic control, dyslipidemia
[59]	16 T1D patients	↑ spatial QRS-T angle	Marker of hypoglycemia, arrhythmia vulnerability	Independent from catecholamine levels and heart rate variability

SD: standard deviation; CVD: cardiovascular risk; LVH: left ventricular hypertrophy; MI: myocardial infarction, T2D: type 2 diabetes; T1D: type 1 diabetes; QTc: QT interval corrected for heart rate; PCA ratio: principal component analysis (PCA) of the ratio of the second to first eigenvalues of the T-wave, HR: heart rate.

**Table 2:** Recent and major studies of the incidence of electrocardiographic abnormalities in cardiac autonomic neuropathy.

Reference	Population/Animals	ECG marker	Clinical significance	Clinical points
Classical ECG markers in CAN and diabetes				

[73]	Zucker Diabetic Fatty rats	↑ R wave amplitude, ↑ QT intervals,↓ HRV	Early diagnosis of CAN, Diabetic Cardiomyopathy	Beneficial effect of aerobic exercise in R wave amplitude
[74]	682 T2D + coronary heart disease	↑ QTc	↑ risk for sudden cardiac death	Idiopathic QT prolongation:5-fold ↑ risk of SCD
[75]	1.226 T1D patients	↓ QTc	↓ incidence of CAN with intensive diabetic treatment	14 years follow-up endpoint
[76]	18 healthy subjects, 30–40 years	↓ PR, ↑ QTc, ↓ T-wave amplitude, ↓ ST	Early diagnosis of CAN, Arrhythmia	severe arrhythmias and “dead-in-bed” syndrome in unrecognized hypoglycemia
[77]	1.720 T2D patients + healthy	QTc > 440 msec, ↓ HRV	↑ mortality	↑ QT dispersion not significant predictor
[78]	100 T1D and T2D patients	↑ QTc	CAN	Association with age, diabetes duration, severity of CAN
[79]	192 T2D patients	↑ QTc, ↑ QT dispersion	12-y CVD risk	Superior to ABI, CAN test for CVD risk
[80]	80 T1D patients	↑ QTc	CAN	Absence of ventricular late potentials in QTc
[81]	8.185 healthy people	↓ HRV and ↑ Heart Rate > 73 bpm	60% ↑ risk of T2D	Independent of CVD disease, age, gender, life and style
[82]	105 T1D and T2D patients	↑ QTc > 440 msec	↑ CAN severity (Ewing score)	Association with age, obesity, hypertension, diabetes duration and control, diabetic treatment
[83]	26 males with diabetes	↑ QTc	↑ 3-year SCD risk in CAN	Independent of age, diabetes duration

Vectorcardiography: the alternative proposition in CAN and diabetes				

[84]	5.781, age ≥ 55 years, 12.7% with diabetes	Spatial QRS-T angle ≥ 75°	4-fold ↑ risk of 4-year CVD and SCD	3-fold ↑ risk of fatal and nonfatal CVD events
[85]	4.173, 14% with diabetes	Spatial QRS-T angle ≥ 45°	50%↑ risk of 7-year incident CVD	50%↑ risk of 7-year total mortality
[56]	6.134, 10% with diabetes	Spatial QRS-T angle ≥ 105°	5-fold ↑ risk of CVD death	2-fold ↑ risk of SCD and total mortality
[86]	142 women, 32% with diabetes	Spatial QRS-T angle ≥ 49°	1.5-fold ↑ risk of CVD events	3-year prospective study
[3]	232 T2D patients	↑ spatial QRS-T angle	↑ incidence of CAN ↑ incidence of Diabetic Cardiomyopathy	Association with HRV (↓parasympathetic tone and ↑ sympathetic tone or sympathovagal imbalance)

CAN: cardiac autonomic neuropathy; T2D: type 2 diabetes; T1D: type 1 diabetes; HRV: heart rate variability; QTc: QT interval corrected for heart rate; SCD: sudden cardiac death; CVD: cardiovascular disease; ABI: ankle-brachialindex.

## References

[1] Schnell O, Otter W, Standl E (2009). The Munich Myocardial Infarction Registry: translating the European Society of Cardiology (ESC) and European Association for the Study of Diabetes (EASD) guidelines on diabetes, pre-diabetes, and cardiovascular disease into clinical practice. *Diabetes care*.

[2] Van Dieren S, Beulens JWJ, Van Der Schouw YT, Grobbee DE, Neal B (2010). The global burden of diabetes and its complications: an emerging pandemic. *European Journal of Cardiovascular Prevention and Rehabilitation*.

[3] Voulgari C, Papadogiannis D, Tentolouris N (2010). Diabetic cardiomyopathy: from the pathophysiology of the cardiac myocytes to current diagnosis and management strategies. *Vascular Health and Risk Management*.

[4] Boudina S, Abel ED (2007). Diabetic cardiomyopathy revisited. *Circulation*.

[5] Theosophy (1939). Ancient landmarks: plato and aristotle. *Theosophy*.

[6] Zoungas S, Patel A (2010). Cardiovascular outcomes in type 2 diabetes: the impact of preventative therapies. *Annals of the New York Academy of Sciences*.

[7] Miller TD, Redberg RF, Wackers FJT (2006). Screening asymptomatic diabetic patients for coronary artery disease: why not?. *Journal of the American College of Cardiology*.

[8] Beller GA (2007). Noninvasive screening for coronary atherosclerosis and silent ischemia in asymptomatic type 2 diabetic patients: is it appropriate and cost-effective?. *Journal of the American College of Cardiology*.

[9] Poirier P, Després J-P, Bertrand OF (2006). Identifying which patients with diabetes should be tested for the presence of coronary artery disease—the importance of baseline electrocardiogram and exercise testing. *Canadian Journal of Cardiology*.

[10] Aneja A, Tang WHW, Bansilal S, Garcia MJ, Farkouh ME (2008). Diabetic cardiomyopathy: insights into pathogenesis, diagnostic challenges, and therapeutic options. *American Journal of Medicine*.

[11] Raev D (1994). Which left ventricular function is impaired earlier in the evolution of diabetic cardiomyopathy? An echocardiographic study of young type I diabetic patients. *Diabetes Care*.

[12] Schannwell CM, Schneppenheim M, Perings S, Plehn G, Strauer BE (2002). Left ventricular diastolic dysfunction as an early manifestation of diabetic cardiomyopathy. *Cardiology*.

[13] Kim TH, Yu SH, Choi SH (2011). Pericardial fat amount is an independent risk factor of coronary artery stenosis assessed by multidetector-row computed tomography: the Korean Atherosclerosis Study 2. *Obesity (Silver Spring)*.

[14] Babcock MJ, Soliman EZ, Ding J, Kronmal RA, Goff DC (2011). Pericardial fat and atrial conduction abnormalities in the Multiethnic Study of atherosclerosis (MESA). *Obesity (Silver Spring)*.

[15] Gómez-Marcos MA, Recio-Rodríguez JI, Rodríguez-Snchez E (2010). Central blood pressure and pulse wave velocity: relationship to target organ damage and cardiovascular morbidity-mortality in diabetic patients or metabolic syndrome. An observational prospective study. LOD-DIABETES study protocol. *BMC Public Health*.

[16] Wiik BP, Larstorp ACK, Høieggen A (2010). Serum uric acid is associated with new-onset diabetes in hypertensive patients with left ventricular hypertrophy: the LIFE Study. *American Journal of Hypertension*.

[17] Barrios V, Escobar C, Calderón A, Echarri R, Barrios S, Navarro-Cid J (2009). Electrocardiographic left ventricular hypertrophy regression induced by an angiotensin receptor blocker-based regimen in hypertensive patients with diabetes: data from the SARA study. *Journal of the Renin-Angiotensin-Aldosterone System*.

[18] Okin PM, Devereux RB, Nieminen MS (2001). Relationship of the electrocardiographic strain pattern to left ventricular structure and function in hypertensive patients: the LIFE study. *Journal of the American College of Cardiology*.

[19] James TN (1998). The variable morphological coexistence of apoptosis and necrosis in human myocardial infarction: significance for understanding its pathogenesis, clinical course, diagnosis and prognosis. *Coronary Artery Disease*.

[20] Beckman JA, Libby P, Creager MA, Libby P, Bonow RO, Mann DL, Zipes DP (2008). Diabetes mellitus, the metabolic syndrome, and atherosclerotic vascular disease. *Braunwald’s Heart Disease: A Textbook of Cardiovascular Medicine*.

[21] Dweck M, Campbell IW, Miller D, Francis CM (2009). Clinical aspects of silent myocardial ischaemia: with particular reference to diabetes mellitus. *British Journal of Diabetes and Vascular Disease*.

[22] Cosson E, Attali JR, Valensi P (2005). Markers for silent myocardial ischemia in diabetes. Are they helpful?. *Diabetes and Metabolism*.

[23] Avignon A, Sultan A, Piot C (2007). Osteoprotegerin: a novel independent marker for silent myocardial ischemia in asymptomatic diabetic patients. *Diabetes Care*.

[24] Davis TME, Fortun P, Mulder J, Davis WA, Bruce DG (2004). Silent myocardial infarction and its prognosis in a community-based cohort of Type 2 diabetic patients: the Fremantle Diabetes Study. *Diabetologia*.

[25] Wackers FJ, Young LH, Inzucchi SE (2004). Detection of ischemia in asymptomatic diabetics (DIAD) investigators: detection of silent myocardial ischemia in asymptomatic diabetic subjects. *Diabetes Care*.

[26] Kumar A, Prineas RJ, Arnold AM (2008). Prevalence, prognosis, and implications of isolated minor nonspecific ST-segment and T-wave abnormalities in older adults cardiovascular health study. *Circulation*.

[27] Stein PK, Sanghavi D, Domitrovich PP, Mackey RA, Deedwania P (2008). Ambulatory ECG-based T-wave alternans predicts sudden cardiac death in high-risk post-MI patients with left ventricular dysfunction in the EPHESUS study. *Journal of Cardiovascular Electrophysiology*.

[28] Aguilar D, Goldhaber SZ, Gans DJ (2004). Clinically unrecognized Q-wave myocardial infarction in patients with diabetes mellitus, systemic hypertension, and nephropathy. *American Journal of Cardiology*.

[29] Pappone C, Santlnelli V (2010). Cardiac electrophysiology in diabetes. *Minerva Cardioangiologica*.

[30] Lloyd-Jones D, Adams RJ, Brown TM (2010). American heart association statistics committee and stroke statistics subcommittee. Heart disease and stroke statistics—2010 update: a report from the American Heart Association. *Circulation*.

[31] Shirani J, Dilsizian V (2010). Screening asymptomatic patients with type 2 diabetes mellitus for coronary artery disease: does it improve patient outcome?. *Current Cardiology Reports*.

[32] Bacci S, Villella M, Villella A (2002). Screening for silent myocardial ischaemia in type 2 diabetic patients with additional atherogenic risk factors: applicability and accuracy of the exercise stress test. *European Journal of Endocrinology*.

[33] Cosson E, Paycha F, Pares J (2004). Detecting silent coronary stenoses and stratifying cardiac risk in patients with diabetes: ECG stress test or exercise myocardial scintography. *Diabetic Medicine*.

[34] Valensi P, Cosson E (2010). It is not yet the time to stop screening diabetic patients for silent myocardial ischaemia. *Diabetes and Metabolism*.

[35] Lyerly GW, Sui X, Church TS, Lavie CJ, Hand GA, Blair SN (2008). Maximal exercise electrocardiography responses and coronary heart disease mortality among men with diabetes mellitus. *Circulation*.

[36] Marwick TH, Hordern MD, Miller T (2009). Exercise training for type 2 diabetes mellitus: Impact on cardiovascular risk: a scientific statement from the american heart association. *Circulation*.

[37] Wackers FJ, Chyun DA, Young LH (2007). Resolution of asymptomatic myocardial ischemia in patients with type 2 diabetes in the detection of ischemia in asymptomatic diabetics (DIAD) study. *Diabetes Care*.

[38] Young LH, Wackers FJ, Chyun DA (2009). Cardiac outcomes after screening for asymptomatic coronary artery disease in patients with type 2 diabetes: the DIAD study: a randomized controlled trial. *Journal of the American Medical Association*.

[39] Cardoso CRL, Salles GF, Deccache W (2003). Prognostic value of QT interval parameters in type 2 diabetes mellitus: results of a long-term follow-up prospective study. *Journal of Diabetes and Its Complications*.

[40] Sawicki PT, Kiwitt S, Bender R, Berger M (1998). The value of QT interval dispersion for identification of total mortality risk in non-insulin-dependent diabetes mellitus. *Journal of Internal Medicine*.

[41] Okin PM, Devereux RB, Lee ET, Galloway JM, Howard BV (2004). Electrocardiographic repolarization complexity and abnormality predict all-cause and cardiovascular mortality in diabetes: the strong heart study. *Diabetes*.

[42] Nelson MR, Daniel KR, Carr JJ (2008). Associations between electrocardiographic interval durations and coronary artery calcium scores: the Diabetes Heart Study. *Pacing and Clinical Electrophysiology*.

[43] Žďárská D, Pelíšková P, Charvát J (2007). ECG body surface mapping (BSM) in type 1 diabetic patients. *Physiological Research*.

[44] Christensen TF, Tarnow L, Randløv J (2010). QT interval prolongation during spontaneous episodes of hypoglycaemia in type 1 diabetes: the impact of heart rate correction. *Diabetologia*.

[45] Kubiak T, Wittig A, Koll C (2010). Continuous glucose monitoring reveals associations of glucose levels with QT interval length. *Diabetes Technology and Therapeutics*.

[46] Giunti S, Bruno G, Lillaz E (2007). Incidence and risk factors of prolonged QTc interval in type 1 diabetes: the EURODIAB prospective complications study. *Diabetes Care*.

[47] Stettler C, Bearth A, Allemann S (2007). QT interval and resting heart rate as long-term predictors of mortality in type 1 and type 2 diabetes mellitus: a 23-year follow-up. *Diabetologia*.

[48] Rossing P, Breum L, Major-Pedersen A (2001). Prolonged QTc interval predicts mortality in patients with Type 1 diabetes mellitus. *Diabetic Medicine*.

[49] van der Linde HJ, Van Deuren B, Somers Y, Loenders B, Towart R, Gallacher DJ (2010). The Electro-Mechanical window: a risk marker for Torsade de Pointes in a canine model of drug induced arrhythmias. *British Journal of Pharmacology*.

[50] Giunti S, Bruno G, Veglio M (2005). Electrocardiographic left ventricular hypertrophy in type 1 diabetes. *Diabetes Care*.

[51] Okin PM, Devereux RB, Harris KE (2007). In-treatment resolution or absence of electrocardiographic left ventricular hypertrophy is associated with decreased incidence of new-onset diabetes mellitus in hypertensive patients: the losartan intervention for endpoint reduction in hypertension (LIFE) study. *Hypertension*.

[52] Lehtinen AB, Daniel KR, Shah SA (2009). Relationship between genetic variants in myocardial sodium and potassium channel genes and QT interval duration in diabetics: the diabetes heart study. *Annals of Noninvasive Electrocardiology*.

[53] Van Huysduynen BH, Swenne CA, Draisma HHM (2005). Validation of ECG indices of ventricular repolarization heterogeneity: a computer simulation study. *Journal of Cardiovascular Electrophysiology*.

[54] van Huysduynen BH, Swenne CA, Bax JJ (2005). Dispersion of repolarization in cardiac resynchronization therapy. *Heart Rhythm*.

[55] Voulgari C, Tentolouris N (2009). Assessment of the spatial QRS-T angle by vectorcardiography: current data and perspectives. *Current Cardiology Reviews*.

[56] Kardys I, Kors JA, Van der Meer IM, Hofman A, Van der Kuip DAM, Witteman JCM (2003). Spatial QRS-T angle predicts cardiac death in a general population. *European Heart Journal*.

[57] Rautaharju PM, Kooperberg C, Larson JC, LaCroix A (2006). Electrocardiographic abnormalities that predict coronary heart disease events and mortality in postmenopausal women: the women’s health initiative. *Circulation*.

[58] Voulgari CH, Tentolouris N, Moyssakis I (2006). Spatial QRS-T angle: association with diabetes and left ventricular performance. *European Journal of Clinical Investigation*.

[59] Koivikko ML, Karsikas M, Salmela PI (2008). Effects of controlled hypoglycaemia on cardiac repolarisation in patients with type 1 diabetes. *Diabetologia*.

[60] Vinik AI, Ziegler D (2007). Diabetic cardiovascular autonomic neuropathy. *Circulation*.

[61] Licht CM, Vreeburg SA, van Reedt Dortland AK (2010). Increased sympathetic and decreased parasympathetic activity rather than changes in hypothalamic-pituitary-adrenal axis activity is associated with metabolic abnormalities. *The Journal of Clinical Endocrinology and Metabolism*.

[62] Maser RE, Mitchell BD, Vinik AI, Freeman R (2003). The association between cardiovascular autonomic neuropathy and mortality in individuals with diabetes a meta-analysis. *Diabetes Care*.

[63] Tesfaye S, Boulton AJM, Dyck PJ (2010). Diabetic neuropathies: update on definitions, diagnostic criteria, estimation of severity, and treatments. *Diabetes Care*.

[64] Pop-Busui R, Evans GW, Gerstein HC (2010). Effects of cardiac autonomic dysfunction on mortality risk in the Action to Control Cardiovascular Risk in Diabetes (ACCORD) trial. *Diabetes Care*.

[65] Calles-Escandón J, Lovato LC, Simons-Morton DG (2010). Effect of intensive compared with standard glycemia treatment strategies on mortality by baseline subgroup characteristics: the action to control cardiovascular risk in diabetes (ACCORD) trial. *Diabetes Care*.

[66] Ziegler D, Rathmann W, Meisinger C, Dickhaus T, Mielck A (2009). Prevalence and risk factors of neuropathic pain in survivors of myocardial infarction with pre-diabetes and diabetes. The KORA Myocardial Infarction Registry. *European Journal of Pain*.

[67] Gandhi RA, Marques JLB, Selvarajah D, Emery CJ, Tesfaye S (2010). Painful diabetic neuropathy is associated with greater autonomic dysfunction than painless diabetic neuropathy. *Diabetes Care*.

[68] Pop-Busui R (2010). Cardiac autonomic neuropathy in diabetes: a clinical perspective. *Diabetes Care*.

[69] Tentolouris N, Voulgari C, Liatis S (2010). Moisture status of the skin of the feet assessed by the visual test neuropad correlates with foot ulceration in diabetes. *Diabetes Care*.

[70] Liatis S, Marinou K, Tentolouris N, Pagoni S, Katsilambros N (2007). Usefulness of a new indicator test for the diagnosis of peripheral and autonomic neuropathy in patients with diabetes mellitus. *Diabetic Medicine*.

[71] Elliott J, Tesfaye S, Chaturvedi N (2009). Large-fiber dysfunction in diabetic peripheral neuropathy is predicted by cardiovascular risk factors. *Diabetes Care*.

[72] Soedamah-Muthu SS, Chaturvedi N, Witte DR (2008). Relationship between risk factors and mortality in type 1 diabetic patients in Europe: the EURODIAB Prospective Complications Study (PCS). *Diabetes Care*.

[73] Van Hoose L, Sawers Y, Loganathan R (2010). Electrocardiographic changes with the onset of diabetes and the impact of aerobic exercise training in the Zucker Diabetic Fatty (ZDF) rat. *Cardiovascular Diabetology*.

[74] Chugh SS, Reinier K, Singh T (2009). Determinants of prolonged QT interval and their contribution to sudden death risk in coronary artery disease: the Oregon sudden unexpected death study. *Circulation*.

[75] Pop-Busui R, Low PA, Waberski BH (2009). Effects of prior intensive insulin therapy on cardiac autonomic nervous system function in type 1 diabetes mellitus: the diabetes control and complications trial/epidemiology of diabetes interventions and complications study (DCCT/EDIC). *Circulation*.

[76] Laitinen T, Lyyra-Laitinen T, Huopio H (2008). Electrocardiographic alterations during hyperinsulinemic hypoglycemia in healthy subjects. *Annals of Noninvasive Electrocardiology*.

[77] Ziegler D, Zental CP, Perz S (2008). Prediction of mortality using measures of cardiac autonomic dysfunction in the diabetic and nondiabetic population: the MONICA/KORA Augsburg Cohort study. *Diabetes Care*.

[78] Pappachan JM, Sebastian J, Bino BC (2008). Cardiac autonomic neuropathy in diabetes mellitus: prevalence, risk factors and utility of corrected QT interval in the ECG for its diagnosis. *Postgraduate Medical Journal*.

[79] Rana BS, Lim PO, Naas AAO (2005). QT interval abnormalities are often present at diagnosis in diabetes and are better predictors of cardiac death than ankle brachial pressure index and autonomic function tests. *Heart*.

[80] Grossman G, Schwentikowski M, Keck FS (2004). Signal-averaged electrocardiogram in patients with insulin-dependent (type 1) diabetes mellitus with and without diabetic neuropathy. *Diabetic Medicine*.

[81] Carnethon MR, Golden SH, Folsom AR, Haskell W, Liao D (2003). Prospective investigation of autonomic nervous system function and the development of type 2 diabetes: the Atherosclerosis Risk in Communities Study, 1987–1998. *Circulation*.

[82] Tentolouris N, Katsilambros N, Papazachos G (1997). Corrected QT interval in relation to the severity of diabetic autonomic neuropathy. *European Journal of Clinical Investigation*.

[83] Ewing DJ, Boland O, Neilson JMM, Gho CG, Clarke BF (1991). Autonomic neuropathy, QT interval lengthening, and unexpected deaths in male diabetic patients. *Diabetologia*.

[84] Kors JA, De Bruyne MC, Hoes AW (1998). T axis as an indicator of risk of cardiac events in elderly people. *Lancet*.

[85] Rautaharju PM, Nelson JC, Kronmal RA (2001). Usefulness of T-axis deviation as an independent risk indicator for incident cardiac events in older men and women free from coronary heart disease (the Cardiovascular Health Study). *American College of Cardiology*.

[86] Triola B, Olson MB, Reis SE (2005). Electrocardiographic predictors of cardiovascular outcome in women: the National Heart, Lung, and Blood Institute-sponsored women’s ischemia syndrome evaluation (WISE) study. *Journal of the American College of Cardiology*.

[87] American Academy of Neurology (1996). Assessment: clinical autonomic testing report of the therapeutics and technology assessment subcommittee of the American Academy of Neurology. *Neurology*.

[88] Kassoff A, Catalano RA, Mehu M (1988). Vitreous hemorrhage and the valsalva maneuver in proliferative diabetic retinopathy. *Retina*.

[89] Rosengård-Bärlund M, Bernardi L, Fagerudd J (2009). Early autonomic dysfunction in type 1 diabetes: a reversible disorder?. *Diabetologia*.

[90] Cabezas-Cerrato J, Gonzalez-Quintela A, Perez-Rodriguez M (2009). Combination of cardiorespiratory reflex parameters and heart rate variability power spectrum analysis for early diagnosis of diabetic cardiac autonomic neuropathy. *Diabetes & Metabolism*.

[91] Freeman R (2006). Assessment of cardiovascular autonomic function. *Clinical Neurophysiology*.

[92] Kempler P, Tesfaye S, Chaturvedi N (2001). Blood pressure response to standing in the diagnosis of autonomic neuropathy: the EURODIAB IDDM Complications Study. *Archives of Physiology and Biochemistry*.

[93] Pop-Busui R, Herman WH, Feldman EL (2010). DCCT and EDIC studies in type 1 diabetes: lessons for diabetic neuropathy regarding metabolic memory and natural history. *Current Diabetes Reports*.

[94] Hoyer D, Maestri R, Teresa La Rovere M, Pinna GD (2008). Autonomic response to cardiac dysfunction in chronic heart failure: a risk predictor based on autonomic information flow. *Pacing and Clinical Electrophysiology*.

[95] Ghuran A, Reid F, La Rovere MT (2002). Heart rate turbulence-based predictors of fatal and nonfatal cardiac arrest (The Autonomic Tone and Reflexes After Myocardial Infarction substudy). *American Journal of Cardiology*.

[96] Heitmann A, Huebner T, Schroeder R, Perz S, Voss A (2011). Multivariate short-term heart rate variability: a pre-diagnostic tool for screening heart disease. *Medical and Biological Engineering and Computing*.

[97] Quattrini C, Jeziorska M, Tavakoli M, Begum P, Boulton AJM, Malik RA (2008). The Neuropad test: a visual indicator test for human diabetic neuropathy. *Diabetologia*.

[98] Tentolouris N, Achtsidis V, Marinou K, Katsilambros N (2008). Evaluation of the self-administered indicator plaster neuropad for the diagnosis of neuropathy in diabetes. *Diabetes Care*.

[99] Lombardi F (2002). Clinical implications of present physiological understanding of HRV components. *Cardiac Electrophysiology Review*.

[100] Voulgari C, Psallas M, Kokkinos A, Argiana V, Katsilambros N, Tentolouris N (2011). The association between cardiac autonomic neuropathy with metabolic and other factors in subjects with type 1 and type 2 diabetes. *Journal of Diabetes and Its Complications*.

[101] Tentolouris N, Liatis S, Moyssakis I (2003). Aortic distensibility is reduced in subjects with type 2 diabetes and cardiac autonomic neuropathy. *European Journal of Clinical Investigation*.

[102] Cruickshank K, Riste L, Anderson SG, Wright JS, Dunn G, Gosling RG (2002). Aortic pulse-wave velocity and its relationship to mortality in diabetes and glucose intolerance: an integrated index of vascular function?. *Circulation*.

[103] Veglio M, Chinaglia A, Cavallo P (2000). The clinical utility of QT interval assessment in diabetes. *Diabetes, Nutrition and Metabolism—Clinical and Experimental*.

[104] Aronson D, Weinrauch LA, D’Elia JA, Tofler GH, Burger AJ (1999). Circadian patterns of heart rate variability, fibrinolytic activity, and hemostatic factors in type I diabetes mellitus with cardiac autonomic neuropathy. *American Journal of Cardiology*.

[105] Bexton RS, Vallin HO, Camm AJ (1986). Diurnal variation of the QT interval—influence of the autonomic nervous system. *British Heart Journal*.

[106] Valensi PE, Johnson NB, Maison-Blanche P, Extramania F, Motte G, Coumel P (2002). Influence of cardiac autonomic neuropathy on heart rate dependence of ventricular repolarization in diabetic patients. *Diabetes Care*.

[107] Ong JJC, Sarma JSM, Venkataraman K, Levin SR, Singh BN (1993). Circadian rhythmicity of heart rate and QTc interval in diabetic autonomic neuropathy: implications for the mechanism of sudden death. *American Heart Journal*.

[108] Kocovic D, Velimirovic D, Djordjevic M, Pavlovic S, Savic D, Stojanov P (1988). Association between stimulated QT interval and ventricular rhythm disturbances influence of autonomic nervous system. *Pacing and Clinical Electrophysiology*.

[109] Soliman EZ, Elsalam MA, Li Y (2010). The relationship between high resting heart rate and ventricular arrhythmogenesis in patients referred to ambulatory 24 h electrocardiographic recording. *Europace*.

[110] Farrell TG, Bashir Y, Cripps T (1991). Risk stratification for arrhythmic events in postinfarction patients based on heart rate variability, ambulatory electrocardiographic variables and signal-averaged electrocardiogram. *Journal of the American College of Cardiology*.

[111] Tamaki S, Yamada T, Okuyama Y (2009). Cardiac Iodine-123 Metaiodobenzylguanidine imaging predicts sudden cardiac death independently of left ventricular ejection fraction in patients with chronic heart failure and left ventricular systolic dysfunction. Results from a comparative study with signal-averaged electrocardiogram, heart rate variability, and QT dispersion. *Journal of the American College of Cardiology*.

[112] Kowalewski MA, Urban M, Florys B, Peczynska J (2002). Late potentials—are they related to cardiovascular complications in children with type 1 diabetes?. *Journal of Diabetes and Its Complications*.

[113] Van Oosterom A, Hiraoka M, Ogawa S, Kodam I, Inoue M, Kasanuki H, Katoh T (2005). Reflections on T waves. *Advances in Electrocardiology*.

[114] Sahu P, Lim PO, Rana BS, Struthers AD (2000). QT dispersion in medicine: electrophysiological Holy Grail or fool’s gold?. *QJM*.

[115] Luo S, Michler K, Johnston P, MacFarlane PW (2004). A comparison of commonly used QT correction formulae: the effect of heart rate on the QTc of normal ECGs. *Journal of Electrocardiology*.

[116] Bravenboer B, Hendriksen PH, Oey LP, Gispen WH, Van Huffelen AC, Erkelens DW (1993). Is the corrected QT interval a reliable indicator of the severity of diabetic autonomic neuropathy?. *Diabetes Care*.

[117] Psallas M, Tentolouris N, Cokkinos A, Papadogiannis D, Cokkinos DV, Katsilambros N (2006). QT dispersion: comparison between diabetic and non-diabetic individuals and correlation with cardiac autonomic neuropathy. *Hellenic Journal of Cardiology*.

[118] Voulgari C, Moyssakis I, Perrea D, Kyriaki D, Katsilambros N, Tentolouris N (2010). The association between the spatial QRS-T angle with cardiac autonomic neuropathy in subjects with Type 2 diabetes mellitus. *Diabetic Medicine*.

[119] Batchvarov V, Kaski JC, Parchure N (2002). Comparison between ventricular gradient and a new descriptor of the wavefront direction of ventricular activation and recovery. *Clinical Cardiology*.

[120] Edenbrandt L, Jakobsson A, Lindvall E, Bitzén PO, Pahlm O (1997). Increased prevalence of large bites in 12-lead vectorcardiograms of diabetic patients. *Journal of Electrocardiology*.

[121] Casis O, Echevarria E (2004). Diabetic cardiomyopathy: electromechanical cellular alterations. *Current Vascular Pharmacology*.

[122] Niwa N, Nerbonne JM (2010). Molecular determinants of cardiac transient outward potassium current (I) expression and regulation. *Journal of Molecular and Cellular Cardiology*.

[123] Billman GE (2009). Cardiac autonomic neural remodeling and susceptibility to sudden cardiac death: effect of endurance exercise training. *American Journal of Physiology*.

[124] Kim M, Oh JK, Sakata S (2008). Role of resistin in cardiac contractility and hypertrophy. *Journal of Molecular and Cellular Cardiology*.

